# Peanut allergy: Beyond the oral immunotherapy plateau

**DOI:** 10.1002/clt2.12046

**Published:** 2021-08-13

**Authors:** Kelly Bruton, Paul Spill, Derek K. Chu, Susan Waserman, Manel Jordana

**Affiliations:** ^1^ Department of Medicine McMaster Immunology Research Centre (MIRC) McMaster University Hamilton Ontario Canada; ^2^ Department of Medicine McMaster University Hamilton Ontario Canada; ^3^ Department of Health Research Methods, Evidence & Impact McMaster University Hamilton Ontario Canada; ^4^ The Research Institute of St. Joe's Hamilton Hamilton Ontario Canada

**Keywords:** food allergy, IgE, oral immunotherapy, peanut

## Abstract

**Background:**

There are a lack of disease‐modifying treatments for peanut allergy, which is lifelong in most instances. Oral immunotherapy has remained at the forefront of prospective treatments, though its efficacy is consistently undermined by the risk of adverse reactions and meager sustained effects.

**Aim:**

This review discusses the current state of oral immunotherapy, its strengths and limitations, and the future of therapeutics for the treatment of peanut allergy.

**Conclusion:**

The persistence of peanut allergy is currently attributed to reservoirs of peanut‐specific memory B cells and Th2 cells, though the cellular and molecular interplay that facilitates the replenishment of peanut‐specific IgE remains elusive. Uncovering these events will prove critical for identification of novel targets as we forge ahead to a new age of peanut allergy treatment with biotherapeutics.

## 1 INTRODUCTION

Food allergies are an increasing global health burden, with prevalence approaching 8% in developed countries.^1^ The standard of care is strict allergen avoidance, where accidental exposures leading to anaphylaxis are treated with emergency epinephrine auto‐injectors.^2^ The lack of disease‐modifying treatments for food allergy is alarming, given that the disease course is often lifelong – as in the case of allergy to peanut (PN) and tree nuts – and can be life‐threatening.^3^, ^4^

## 2 EFFICACY OF PEANUT ORAL IMMUNOTHERAPY

Oral immunotherapy (OIT) has been in the spotlight as an emerging treatment for PN allergy, though its core methodology has not changed since its original description in 1908.^5^ OIT involves the introduction of a particular allergen at minute oral doses (1–50 mg) to establish a starting dose below the threshold of reactivity. Subsequently, patients enter a dose escalation phase that occurs over a period of months until reaching predefined endpoints, such as a long‐term maintenance dose of >400 mg PN. The primary assessment of OIT success is clinical desensitization, which is defined as an increased threshold of allergen consumption during a supervised oral food challenge (OFC). OIT has proven efficacious with regard to the induction of desensitization,^6^ with two recent clinical trials reporting 67.2% and 85% success rates.^7^, ^8^ Notably, OIT trials often differ in their inclusion criteria (e.g., age) and protocol (e.g., maintenance dosage and follow‐up schedule), perhaps, providing reason as to the variance in success rates. The underlying immunological mechanisms that support the induction of a desensitized state are ambiguous. Generally, desensitization is attributed to decreased IgE, increased IgG4 and regulatory T cells (Tregs), and Th2 cell exhaustion, though many of these contentions are drawn from correlations with limited or no causative proof.

The therapeutic strategy post‐desensitization remains unresolved and, with the relatively poor compliance, lifelong treatment seems unlikely. A growing body of literature suggests that some patients exhibit lasting clinical benefits following cessation of OIT. This phenomenon has been termed sustained unresponsiveness (SU) and refers to the continuance of a desensitized state (assessed by OFC) following discontinuation of the maintenance dose. SU has been almost exclusively assessed at 1–2 months post‐cessation, where half or more of desensitized patients pass an in‐clinic supervised OFC. However, beyond this arbitrary 1–2 months timeframe the prevalence of SU declines, with the POISED study reporting only 13% SU (vs. 4% on placebo) at 1 year post‐OIT.^8^ Again, the biological events that enable a period of clinical tolerance and, ultimately, undermine SU are poorly described.

## 3 SAFETY OF PEANUT ORAL IMMUNOTHERAPY

Recently, evidence has emerged that critically appraises the safety of OIT. In the process of allergen up‐dosing and maintenance, numerous adverse reactions can occur. Reactions involve mild to severe gastrointestinal, respiratory, and/or dermatological symptoms, with the most severe unintended effect being anaphylaxis. The safety profile of PN‐OIT has been systematically reviewed in the PACE study,^9^ where it was established that OIT increased the risk of anaphylaxis, epinephrine use, and other allergic symptoms compared to the current standard of care (strict avoidance).^9^ However, as noted by Eiwegger et al.*,* ^10^ there are issues that remain to be clarified. For example, the PACE study did not distinguish between those adverse events that were the result of treatment versus those that resulted from the accidental exposures. While this could enhance the assessment of treatment‐related risk, it is not intuitive how this distinction could be ascertained given that patients take the treatment daily. Furthermore, PN allergy is lifelong in most patients and the efficacy of OIT, understood as desensitization, requires continued administration of PN. Therefore, an assessment of the safety profile of PN OIT over long‐term treatment, not only during the induction/initial maintenance phase, is still needed. The same logic applies to the assessment of quality of life (QoL) over the long term as most adverse effects emerge during the up‐dosing phase.^11^ OIT is no different than most other treatments in that its implementation must be decided after a comprehensive risk‐benefit evaluation. Ultimately, understanding of the findings pertaining to the efficacy, safety and impact on QoL advocates for informed shared decision‐making between patients, their families, and health care professionals when considering OIT, and safer management approaches to its implementation.^12^

OIT has been a dominant theme in the field of food allergy research. However, the same core approach has been researched and implemented for over 20 years^5^, ^13^, ^14^ through endless protocol modifications and arguably, a plateau as to what OIT can and cannot do has been reached. The path forward to the discovery of disease‐modifying therapies is hampered by our limited understanding of the cellular and molecular mechanisms that perpetuate IgE responses to food allergens. A progression towards the use of targeted biotherapeutics with the potential to modify the underlying disease process requires remedying this knowledge gap.

## 4 IMMUNOLOGICAL MEMORY IN FOOD ALLERGY

In IgE‐mediated disease, IgE levels have been shown to decline in periods of non‐allergen exposure. In humans affected by seasonal allergic rhinitis, there is documented evidence of this decline, where IgE titers are cyclical coinciding with allergen exposure.^15^ Moreover, IgE titers specific to the fish parasite, *Anisakis* spp., drastically decline following 10 months without fish consumption in *Anisakis* spp.‐allergic humans.^16^ This is difficult to observe in food‐allergic individuals due to the high incidence of accidental exposures^17^; however, experimental models of food allergy in mice, where allergen exposure can be precisely controlled, support this notion.^18^ As the half‐life of IgE is <72 hours in serum, this evidence would suggest that declining IgE titers are, in fact, due to a loss of IgE^+^ plasma cells (PCs). Similarly, IgE‐secreting cells in peripheral blood of food‐allergic individuals were discovered to have an immature transcriptional profile with downregulated expression of plasma cell survival genes.^19^ Thus, a quiescent cell capable of regenerating the plasma cell pool is the probable reservoir of IgE responses.

**FIGURE 1:**
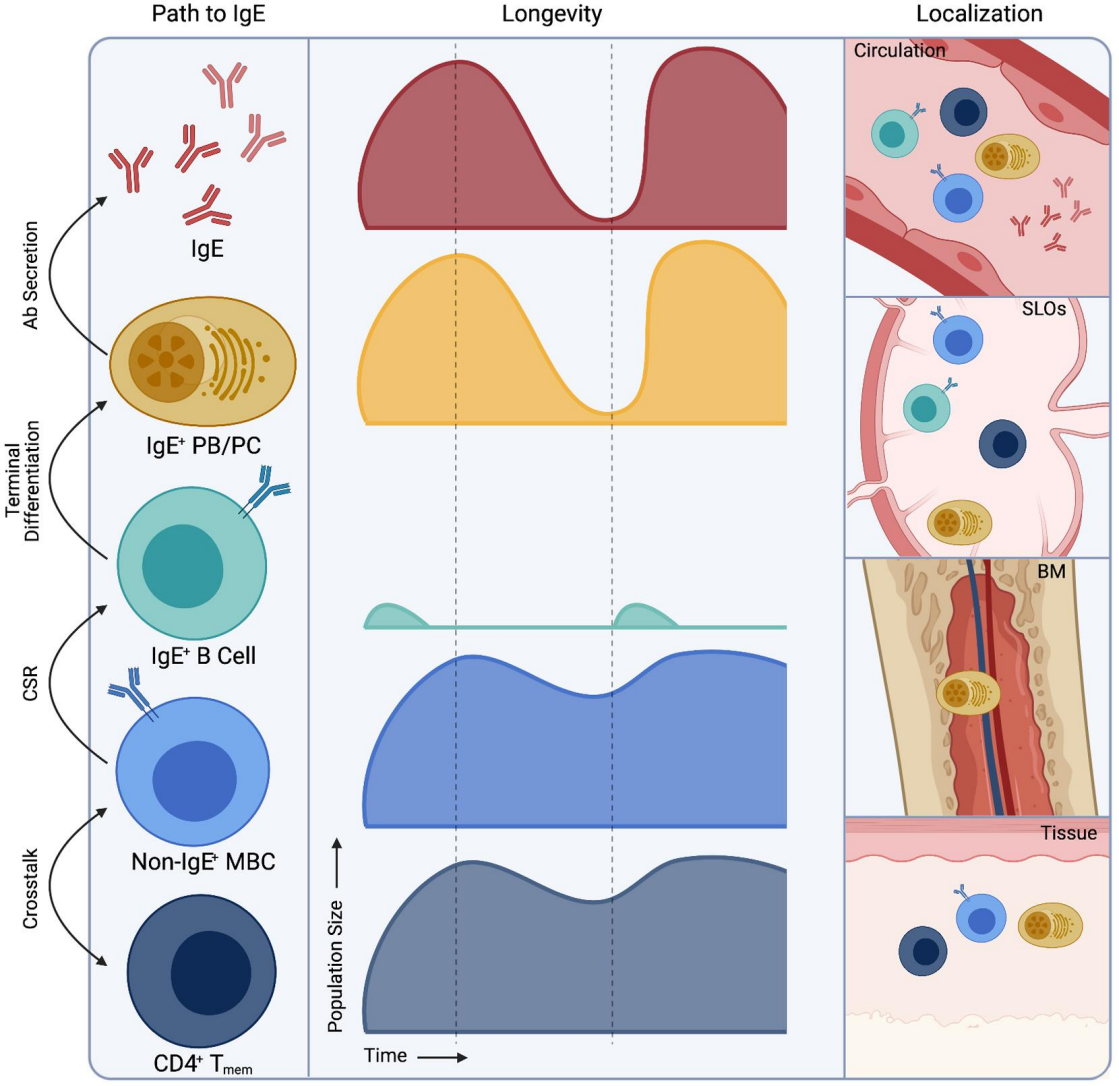
Key features of immunological memory mediating lifelong food allergies. Ab, antibody; CSR, class switch recombination; SLOs, secondary lymphoid organs; BM, bone marrow; PB, plasmablast. Space between dashed lines represents period without allergen exposure

In this regard, memory B cells (MBCs) have been a focal point of recent investigations on the maintenance of food allergy. Through the application of advanced flow cytometry strategies^20^ and single‐cell RNA‐sequencing,^19^ the extreme rarity of IgE^+^ MBCs has been described in human peripheral blood mononuclear cells of allergic subjects. This does not, however, discount the existence of IgE^+^ MBCs/PCs at secondary lymphoid or non‐lymphoid tissue sites. In gastrointestinal biopsies from subjects with PN allergy, Hoh et al.^21^ identified reservoirs of IgE^+^ PCs (IgE^+^ CD138^+^) in the stomach and duodenum, but did not detect IgE^+^ B cells (IgE^+^ CD138^‐^ with small B lymphocyte morphology). The longevity of gastric and duodenal IgE^+^ PCs was not described, though identification of non‐IgE isotypes within IgE clonal lineages at the same tissue sites suggests local IgE class switch recombination.^21^ Most frequently, IgE class switch recombination occurs sequentially, where B cells express one or more intermediate isotypes with IgG1 as the dominant intermediary.^22^ Adoptive transfer of IgG1^+^ MBCs and *IL4‐*transcribing CD4^+^ T cells from Th2‐immunized mice have been shown to drive IgE responses in recipient mice, demonstrating that IgG1^+^ MBCs are sufficient for the perpetuation of IgE responses.^23^ MBCs, however, require crosstalk with CD4^+^ T cells to undergo PC differentiation. A specific subset of Th2‐polarized CD4^+^ T cells, termed “Th2A” cells, has been proposed to drive allergic responses.^24^ Wambre et al.^24^ demonstrated that this CD4^+^ T cell subset uniquely expands and contracts in pollen‐allergic individuals, concordant with on‐ and off‐season allergen peaks. Moreover, in patients achieving clinical desensitization following a 20‐week PN‐OIT regimen, the Th2A cell subset declined, but remained at detectable levels.^24^ This residual population may represent a reservoir of allergen‐specific CD4^+^ T cells capable of subverting SU. An overview of immunological memory to food allergens is provided in Figure 1.

## 5 FUTURE OF THERAPEUTICS

**FIGURE 2:**
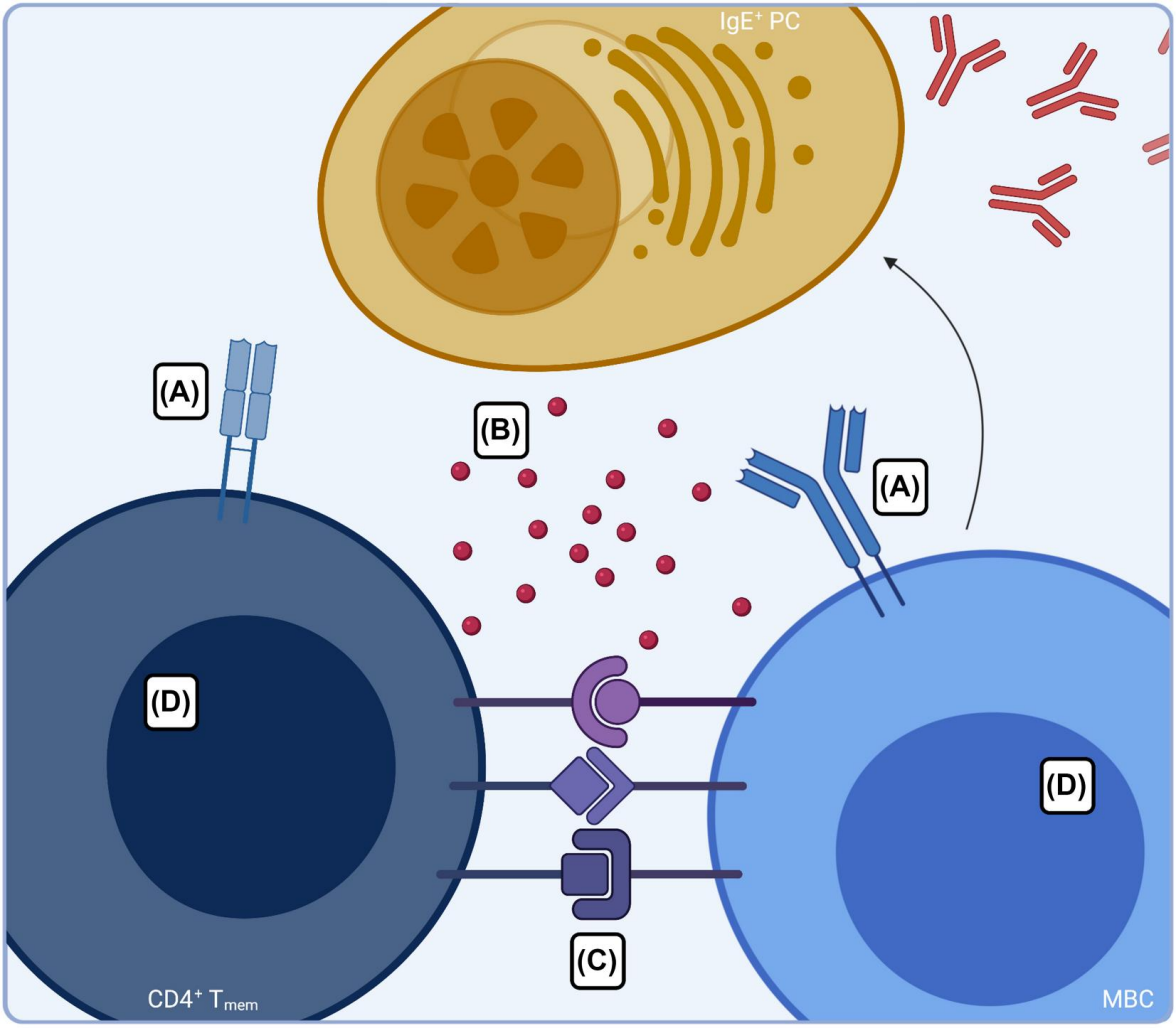
Potential biologic targets to disarm allergic recall responses. (A) Elimination and/or inhibition of allergen‐specific lymphocytes utilizing therapeutics that engage with allergen‐specific T cell or B cell receptors. (B) Inhibition of allergic recall responses with therapeutics targeting key cytokines/chemokines and/or their cell‐surface receptors. (C) Inhibition of allergic recall responses through blockade of co‐stimulatory interactions. (D) Harnessing plasticity of allergen‐specific cells to reprogramming the pathogenic lymphocytes

The current understanding on the maintenance of allergy posits both IgG1^+^ MBCs and Th2A as direct targets for biotherapeutics. In recent years, the field of oncology has seen the rise of approaches, such as CAR‐T cells among others, aimed towards the destruction of malignant cells. Such approaches could theoretically be adopted for food allergy, with an aim to specifically kill pathogenic allergen‐specific cell repertoires (Figure 2A). However, while the off‐target effects of cytotoxic therapies are often accepted in patients suffering from cancer given their potential to improve lifespan, these types of therapies may need to see significant refinement before they could be applied to diseases that are typically of a more benign nature, such as allergy. Moreover, it is unclear if deletion of T cells or B cells alone would be sufficient for the resolution of food allergy. For example, with deletion of allergen‐specific B cells, allergen‐specific CD4^+^ T cells may be sufficient to initiate de novo B cell responses, or vice versa.

Alternatively, the critical molecules that facilitate IgE recall responses by allergen‐specific MBCs and CD4^+^ T cells may be an effective target for biotherapeutics (Figure 2B–C). Our current understanding of IgE and Th2 biology highlights IL‐4, IL‐5, IL‐9, and IL‐13, as well as co‐stimulatory molecules, such as CD40L, as key contributors to the recall response. There is already evidence for the potential of therapies targeted towards interrupting signaling by these molecules, especially IL‐4 and IL‐13, in other atopic diseases. For example, a human anti‐IL‐4R*α* monoclonal antibody which interrupts signaling of both IL‐4 and IL‐13 has demonstrated convincing efficacy in controlling moderate‐to‐severe atopic dermatitis,^25^ allergic asthma,^26^ and nasal polyps.^27^

In food allergy, IL‐4 is critically involved in allergic sensitization and promotes IgE class switching.^28^ The short‐ and long‐term impact of IL‐4/IL‐13 blockade remain unclear in the context of established food allergy. Given that IgE and IgE‐secreting cells are transient, impeding their replenishment through the blockade of signaling molecules such as IL‐4 and IL‐13 may prevent the regeneration of IgE against foods. Moreover, following neutralization of pro‐Th2 molecules, subsequent exposure to food allergens may either alter the profile of pathogenic B and T cells or allow for the expansion of competing and/or tolerogenic cell types (Figure 2D). The advent of deep learning, particularly at the single‐cell level, now provides an unprecedented ability to comprehensively interrogate the molecular profile of allergen‐specific lymphocytes, the ontogeny of IgE‐secreting cells in a memory response, and the involved cellular networks. Elucidation of these processes is critical to both revealing new biotherapeutic targets, as well as assessing whether there is disease‐modifying potential in our current therapies. One example of this is derived from our own work in which we employed single‐cell RNA‐sequencing to elucidate transcriptomic profile of human PN‐reactive B and T cells and the impact of anti‐IL‐4R*α* on the recall response. PN‐reactive B and T cells were found to have an IL‐4‐responsive phenotype and application of anti‐IL‐4R*α* demonstrated a critical requirement of IL‐4/IL‐13 signaling for secondary IgE responses.^29^ Remarkably, the aborted IgE response via IL‐4R*α* blockade in vivo was sustained even following clearance of anti‐IL‐4R*α*, proposing that the pathogenic Th2 program retains sufficient plasticity enabling its reprogramming.^29^

To overcome the plateau in food allergy treatment and advance to the “future of therapeutics” proposed above, we foresee at least three immediate *Next Steps:* (1) Elucidate the molecular requirements for recall responses to allergens. Knowledge of the critical molecular interplay is necessary to identify novel therapeutic targets, above and beyond IL‐4/IL‐4R*α*. (2) Characterize the tissue localization of allergen‐specific memory lymphocytes. Most human food allergy research carried out to date has utilized blood samples. While informative, these data are limited in their biological applicability due to exclusion of a potentially key reservoir of tissue‐resident lymphocytes. Moreover, this may inform important considerations as to the route of therapeutic delivery, as systemically administered therapeutics (e.g., dupilumab) may not provide adequate efficacy at tissue sites. (3) Evaluate the plasticity of allergen‐specific B and T cells. Whether pathogenic allergen‐specific lymphocytes retain a malleable phenotype will inform as to the requirement of lifelong versus transient therapeutic regimens.

## 6 CONCLUSION

Herein, we have summarized the current state of knowledge on OIT, described some of the key cells involved in the perpetuation of allergy, and highlighted the advent of a new era of research and treatment in allergic disease. The way forward will center around targeted biotherapeutics. The practical implementation of these new therapies, however, may require marriage with the strategies, such as OIT, that have been developing for over decades. Strict allergen avoidance may be necessary while biotherapeutics offer comfort in raising the threshold of reactivity in the case of accidental exposure, or while waiting for the gradual tapering of existing IgE and IgE‐secreting cells. OIT may be instrumental in administering significant doses of allergen in the safest way possible in order to incite the immune system to reprogram pathogenic cell types or expand cells that will eventually maintain lifelong tolerance in a previously food‐allergic patient. Optimizing the safety and efficacy of OIT is sensical, given that its clinical benefits are appealing for some patients. However, OIT at its current stage does not appear to be a cure for food allergy. The lack of disease‐modifying therapies beckons towards gathering further fundamental insights into the cellular and molecular mechanisms that drive and perpetuate food allergy, and the subsequent implementation of targeted therapies with curative potential.

## CONFLICT OF INTEREST

The authors declare no conflicts of interest.

## AUTHORS CONTRIBUTIONS

Kelly Bruton, Paul Spill, Derek K. Chu, Susan Waserman and Manel Jordana. conceived, wrote, and edited the manuscript.

## CONSENT FOR PUBLICATION

All authors consent to the publication of this review.
